# Mental health and substance use problems among adolescents in Lesotho: Prevalence, access to care, and association with lifestyle factors

**DOI:** 10.1111/jora.70062

**Published:** 2025-08-26

**Authors:** Natalie E. Johnson, Irene Falgas‐Bague, Frédérique Chammartin, Felix Gerber, Tristan T. Lee, Ravi Gupta, Relebohile Mjadu, Jonase Nthunya, Moleboheng Mokebe, Retsélisitsoe Makabateng, Mamoronts’ane P. Sematle, Pearl Letsoela, Niklaus D. Labhardt, Alain Amstutz, Jennifer M. Belus

**Affiliations:** ^1^ Division of Clinical Epidemiology, Department of Clinical Research University Hospital Basel Basel Switzerland; ^2^ University of Basel Basel Switzerland; ^3^ Department of Epidemiology and Public Health Swiss Tropical and Public Health Institute Allschwil Switzerland; ^4^ SolidarMed Maseru Lesotho; ^5^ Government of Lesotho Ministry of Health and Social Welfare Maseru Lesotho; ^6^ Oslo Centre for Biostatistics and Epidemiology Oslo University Hospital Oslo Norway; ^7^ University of Bristol Population Health Sciences Bristol UK

**Keywords:** adolescents, anxiety, depression, PTSD, substance use, sugar‐sweetened beverages

## Abstract

Adolescence is a developmental window in which mental health symptomatology and other health behaviors can set lifelong trajectories. Yet, adolescent development research in rural areas of low‐resource communities is severely lacking. Conceptualized in the Adolescent Well‐Being Framework, where diet and activity form a core domain of well‐being, we examined two modifiable risk behaviors (sugar‐sweetened beverage consumption and physical activity) and their associations with mental health and substance use problems among 1351 adolescents aged 10–17 years (51% girls) in a rural setting in Lesotho, southern Africa. Standardized screening measures showed at least one clinically relevant mental health problem among 3% of the overall sample and 5% among 15–17 year‐olds. Among those in the overall sample meeting these criteria, 45% recognized a need for help, yet only 17% obtained any care, leaving an 83% treatment gap. After adjusting for age and sex, any sugar‐sweetened beverage consumption was associated with having at least one mental health symptom or recent substance use and with clinically relevant mental health and substance use problems. Physical activity showed no significant associations. Although the overall prevalence of clinically relevant mental health and substance use problems was relatively low, low levels of problem awareness suggest a need for adolescent‐appropriate demand‐side interventions for early treatment. Future research may explore possible indigenous protective factors underlying the low prevalence and clarify the pathway between health behaviors and mental health problems.

## INTRODUCTION

Adolescence, marked by rapid physical, emotional, and psychological changes, is a critical developmental phase susceptible to the impacts of adversity, social disparities, and lifestyle choices (Patel et al., 2007; Sisk & Gee, 2022). According to the World Health Organization (WHO), 1 in 7 youth live with a mental disorder globally (WHO, 2021). Adolescence is also the peak time for initiation of substance use, with potential for disruption of key periods of cognitive, emotional, and psychosocial development, leading to long‐term adverse health and social outcomes (Degenhardt et al., 2016; Hall et al., 2016). Seventy‐five percent of all mental health and substance use disorders emerge by the age of 25 (Kessler et al., 2005), underscoring the importance of evaluating prevalence, risk factors, and treatment uptake for mental health and substance use problems during the adolescent period.

Globally, population‐based estimates of mental health and substance use problems among younger adolescents aged 10–14 years range between 0.3% and 14% (Institute of Health Metrics and Evaluation, 2021). Among middle and older adolescents aged 15–19 years globally, prevalence rates of these problems range between 3% and 19% (Bie et al., 2024; Kieling et al., 2024; United Nations Office on Drugs and Crime [UNODC], 2024; WHO, 2018). Data generally suggest that mental health symptoms and substance use problems increase throughout the adolescent period, although there are gender‐specific differences (de Looze et al., 2025; Myhr et al., 2024). For example, depressive symptoms increase rapidly from early to mid‐adolescence for girls, whereas boys typically show a later and more gradual increase (Griffith et al., 2021). Regarding substance use, more boys tend to show higher rates of substance use during mid‐to‐late adolescence, as compared to girls (Moor et al., 2020).

Although population‐based studies estimate that 6%–28% of African adolescents experience mental health or substance use problems (Cortina et al., 2012; Jakobsson et al., 2024; Jörns‐Presentati et al., 2021; Ogundipe et al., 2018; UNODC, 2024; WHO, 2018), most data come from relatively high‐resourced and urban contexts. Marfo (2016) argues that a truly global science of adolescent development requires representativeness in (1) samples, (2) measured constructs and their cultural meanings, and (3) worldviews, idea systems, and ethnotheories that shape conceptions, representations, and meanings of developmental phenomena. The first pillar remains the most conspicuous gap: rural, subsistencefarming communities—which comprise the majority of the African population (World Bank, 2024)—are often missing or of poor quality in large‐scale epidemiological surveys (Seidler et al., 2025). Progress on the second pillar is emerging through targeted translations and psychometric validations of standard instruments for African youth (e.g., see Carvajal‐Velez et al., 2023; Nyongesa et al., 2022), while work examining local worldviews is growing but still fragmented across diverse cultural conceptions of distress and resilience (Haroz et al., 2017; Heim et al., 2022; Rotzinger et al., 2025). Because reliable prevalence estimates from representative samples provide the empirical foundation upon which culturally attuned measures and theories can be built, collecting data using representative samples that include rural populations is a logical first step.

Lesotho is a primarily rural, landlocked country of 2.3 million inhabitants in southern Africa (Ministry of Health Lesotho & United States Agency for International Development [USAID], 2024). Like in many African countries, there is a large adolescent population in Lesotho, with youth aged 10 to 19 years accounting for more than one fifth of the population (Ministry of Health Lesotho & USAID, 2024). Recent surveys indicate that adolescents in Lesotho face challenges with mental health and substance use, such as mental distress and binge drinking (Picchetti et al., 2022). While we are not aware of available estimates of treatment cascades for these problems among adolescents in Lesotho, recent estimates from a population‐based survey with adults suggest that 62% of adults with clinical levels of mental health symptoms and 89% with unhealthy substance use were unaware of their need for treatment. Only 20% had accessed treatment for their mental health condition, while only 5% accessed care for their substance use problems (Fernández, Yoon, et al., 2024). An important first step to providing culturally appropriate adolescent‐centered care is to therefore understand the awareness and treatment cascade for adolescent mental health and substance use problems in Lesotho.

Numerous factors increase the risk of mental health and substance use problems during adolescence, including adverse childhood experiences (e.g., bullying, neglect, violence), urban stressors, a negative and stressful family climate (e.g., high conflict, parental mental illness) (Blakemore, 2019), higher family income, peer substance use, and genetics (Bozzini et al., 2020; Morojele et al., 2021). Because most of these risk factors are either fixed (e.g., genetics) or lie outside an adolescent's sphere of influence (e.g., early adversity, household wealth), researchers increasingly focus on modifiable influences—behaviors that young people themselves can actively change or that policy can feasibly target during adolescence. To target modifiable factors, Ross et al.'s (2020) Adolescent Well‐Being Framework categorizes the prerequisites for adolescent thriving into five intersecting domains: (1) good health and nutrition, (2) connectedness and positive social contribution, (3) safety and a supportive environment, (4) learning, competence, education, and employability, and (5) agency and resilience. Within the first domain, sub‐components such as physical activity and a healthy diet are highlighted as everyday health behaviors which adolescents can directly modify on their own. Evidence from high‐income settings links low physical activity and high sugar‐sweetened beverage intake with worsened mental health among adolescents (Pabayo et al., 2016; Rodriguez‐Ayllon et al., 2019). However, comparable data from African settings remains limited, with one study of physical activity in Ghana showing a similar association between mental health and physical activity (Asare & Danquah, 2015), and no studies with adolescents in the region examining the link between mental health and sugar‐sweetened beverage consumption. Globally, sugar‐sweetened beverage consumption is rising among adolescents, and rural‐to‐urban transitions limit daily physical activity (Baird et al., 2025). Consumption of sugar‐sweetened beverages is rising more rapidly among adolescents in the African region than in any other region (Lara‐Castor et al., 2024), while lifestyle transitions driven by urbanization, mechanization of manual labor, and increased sedentary recreation are contributing to declining levels of physical activity (Boakye et al., 2023; Ojiambo et al., 2012). Together, these shifts emphasize the growing relevance of these two modifiable lifestyle behaviors in the African context.

The present study expands what is known about adolescent mental health and substance use in low‐resource settings using a population‐based sample of adolescents aged 10 to 17 in the predominantly rural country of Lesotho. The goals of this study were to (1) describe the prevalence of depression, anxiety, suicidal ideation, trauma, post‐traumatic stress, and substance use (tobacco, alcohol, and cannabis) for the overall sample and by adolescence stage and sex; (2) describe awareness of the need for, and access to, mental health and substance use care; and (3) examine the associations between modifiable lifestyle factors of sweetened beverage consumption and physical activity with mental health and substance use problems.

## METHODS

### Procedures

This cross‐sectional, population‐based survey was embedded in the Community‐Based Chronic Care in Lesotho (ComBaCaL) survey, which took place in Mokhotlong and Butha Buthe districts between November 2021 and August 2022. The overall goal of the ComBaCaL survey was to assess the prevalence of noncommunicable diseases among adolescents and adults. Details of the sampling and survey methodology have been published elsewhere (González Fernández et al., 2023). Briefly, two‐stage cluster sampling was utilized, with the population clusters (villages) as primary sampling units and household members as secondary sampling units. From a list containing 785 clusters with at least 30 households each, 120 clusters were randomly selected (60 in each district), stratified by settlement type (urban vs. rural) and accessibility with respect to a catchment health facility (hard‐to‐reach vs. easy‐to‐reach areas). Settlement type was included as a stratification factor to ensure both urban and rural contexts were represented in the sample.

The study's implementing partner was a local nonprofit organization, which has operated in these districts alongside the Ministry of Health for 13 years, primarily within the healthcare system. The organization has a trusted reputation and is well‐respected in the communities where the study took place. When approaching a community to participate in the study, local team members working at the partner organization approached the village chief to arrange a community meeting, where community members could learn about the study and its procedures. Upon arrival at a cluster, village chiefs provided verbal consent. All households in a sampled cluster were eligible if consent was provided by the head of household. Age and sex were then enumerated for all members of the household. Household members ≥10 years old were then randomly selected by an algorithm based on age, sex, and settlement (rural vs. urban), programmed in the Open Data Kit data collection tool (Brunette & Hartung, 2023). Specifically, the algorithm maintained separate enrollment quotas for males and females within each predefined age stratum, ensuring a balanced sex distribution across all age groups. Consented and enrolled study participants were subsequently interviewed in a private space in or near the participant's household. Interviews lasted approximately one and a half hours and were conducted in Sesotho.

Prior to the enrollment of any study participants, ethics clearance was obtained from the Lesotho Ministry of Health Research and Ethics Committee (ID 139‐2021). Clarification of responsibility was provided by the Ethics Committee of Northwest and Central Switzerland (ID AO_2021‐00056), meaning that the study was reviewed in Switzerland for scientific integrity, however the Swiss ethics board did not take responsibility for approval because no data were collected within its area of jurisdiction.

### Participants

Among 3919 sampled households, 109 declined to consent and 325 were ineligible because no eligible participants were present when the study team visited, yielding 3485 participating households (Fernández, Firima, et al., 2024). Of these, 936 households contained at least one adolescent aged 10–17 years. Written consent was obtained from a caregiver and verbal assent from each adolescent. A total of 1351 adolescents were enrolled (*M*_age_ = 14.0 years, *SD* = 2.1, range = 10–17); 50.8% were female, 59.5% lived in urban or peri‐urban settlements, and 49.3% resided in households that experienced food shortage in the previous month. All enrolled adolescents completed the full assessment battery. Descriptive characteristics for the 1351 enrolled adolescents are presented in Table 1. Additional demographic details disaggregated by sex appear in Table S1.

**TABLE 1:** Sample characteristics overall and by age group.

Characteristic	Total	Age 10–14	Age 15–17
	*N* = 1351 (%)	*n* = 854 (63.2)	*n* = 497 (36.8)
Sex			
Male	665 (49.2)	423 (49.5)	242 (48.7)
Female	686 (50.8)	431 (50.5)	255 (51.3)
Setting			
Urban	804 (59.5)	504 (59.0)	300 (60.4)
Rural	547 (40.5)	350 (41.0)	197 (39.6)
Highest level of education completed			
No schooling	11 (0.8)	6 (0.7)	5 (1.0)
Primary school	811 (60.0)	662 (77.5)	149 (30.0)
Secondary school	525 (38.9)	186 (21.8)	339 (68.2)
Tertiary school^a^	4 (0.3)	0 (0)	4 (0.8)
Fruit beverage consumption in the past week			
None	781 (57.8)	475 (55.6)	306 (61.6)
Once or twice	413 (30.6)	269 (31.5)	144 (29.0)
Three to four times	93 (6.9)	66 (7.7)	27 (5.4)
Five or more times	63 (4.6)	43 (5.0)	20 (4.0)
Missing	1 (0.1)	1 (0.1)	0 (0)
Soda consumption in the past week			
None	1007 (74.5)	639 (74.8)	368 (74.0)
Once or twice	297 (22.0)	193 (22.6)	104 (20.9)
Three to four times	35 (2.6)	17 (2.0)	18 (3.6)
Five or more times	11 (0.8)	4 (0.5)	7 (0.8)
Missing	1 (0.1)	1 (0.1)	0 (0)
Physical activity level			
Low	681 (50.4)	492 (57.6)	189 (38.0)
Moderate	497 (36.8)	274 (32.1)	223 (44.9)
High	147 (10.9)	67 (7.8)	80 (16.1)
Missing	26 (1.9)	21 (2.5)	5 (1.0)
Self‐reported HIV status			
Positive	17 (1.2)	7 (0.8)	10 (2.0)
Negative	543 (40.2)	285 (33.4)	258 (51.9)
Unknown	790 (58.5)	560 (65.6)	229 (46.1)
Missing	2 (0.1)	2 (0.2)	0 (0)
Food shortage in the past month	666 (49.3)	424 (49.6)	242 (48.7)
Missing	3 (0.2)	3 (0.4)	0
Number of people in the household, Med (IQR)	3 (3–5)	4 (3–6)	3 (3–5)
Missing	11 (0.8)	8 (0.9)	3 (0.6)

Abbreviations: Med, median; IQR, interquartile range.

^a^ For adolescents aged 16–17 years, “tertiary school” reflects completion of a vocational training program. The survey question was phrased, “What is the highest level of education you have completed?” All respondents who selected this option were 16 or 17 years old.

### Measures

Trained study team members assessed participant demographics, mental health, substance use, physical activity, sweetened beverage consumption, as well as awareness of a need for, and access to, mental health or substance use care. Measurement tools were translated from English to Sesotho following standardized translation procedures. The initial translation and subsequent back‐translation were conducted independently by two team members fluent in both English and Sesotho. The initial and back‐translated versions of the tools were then compared by a third bilingual team member and finalized via reconciliation between the three team members.

#### Socio‐demographics

Sex, age, and highest level of education were collected at the individual level. Participants were grouped into age categories of those 10–14 and 15–17 years old, which corresponds to early and middle adolescence, respectively (WHO, 2006). At the household level, we collected information as part of the Demographic and Health Survey wealth index questionnaire (USAID, 2014). Specifically, we asked about the number of people living in the household and whether the household experienced any food shortage in the past month. The latter item was used to approximate household wealth with the following question, “In the past 4 weeks, did you worry that your household would not have enough food?”

#### Depression

Symptoms of depression were measured with the 9‐item Patient Health Questionnaire (PHQ‐9; Kroenke et al., 2001). Items inquire about the experience of the nine hallmark symptoms of depression over the past two weeks, including having little interest or pleasure in doing things. Each item is rated on a 4‐point scale (0 = *Not at all*, 1 = *Several days*, 2 = *More than half the days*, 3 = *Nearly every day*); summed scores range from 0 to 27. Scores of 0–4 indicate minimal, 5–9 mild, and ≥10 clinically relevant depression symptoms (Kroenke et al., 2001; Moriarty et al., 2015). This measure has been used previously with adults in Lesotho (Cerutti et al., 2016; Fernández, Yoon, et al., 2024) and has been validated with adolescents in South Africa using the standard cut‐off score of ≥10 to predict clinical risk for depression (Marlow et al., 2023). The Cronbach's alpha of this measure in the current sample was .75.

#### Suicidality

Suicidal ideation was assessed with the final item of the PHQ‐9, which asks about thoughts of being better off dead or hurting oneself in some way. Responses of ≥1 indicated the presence of suicidality. This item has been used previously among Botswanan youth to assess thoughts of self‐harm (Brooks et al., 2023).

#### Anxiety

Anxiety symptoms were measured with the 7‐item Generalized Anxiety Disorder Screener (GAD‐7; Spitzer et al., 2006). Symptoms measured are the hallmark symptoms of anxiety over the last two weeks, such as not being able to stop or control worrying. Items are scored 0 = *Not at all*, 1 = *Several days*, 2 = *More than half the days*, 3 = *Nearly every day*; total scores range from 0 to 21. Scores of 0–4 denote minimal, 5–9 mild, and ≥10 clinically relevant anxiety symptoms (Spitzer et al., 2006). A South African validated cut‐off score of ≥6 was applied in the current study to signify clinically relevant anxiety symptoms, as this score was found to have the highest sensitivity and specificity to discriminate South African youth with clinical levels of anxiety (Marlow et al., 2023). This measure has been used previously with adults in Lesotho (Fernández, Yoon, et al., 2024; Marlow et al., 2022) and validated for use with South African adolescents. Cronbach's alpha of this measure in the current sample was .74.

#### Trauma and post‐traumatic stress

Lifetime trauma and subsequent symptoms of post‐traumatic stress disorder (PTSD) were assessed using the 5‐item Primary Care Post‐Traumatic Stress Disorder Screener (PC‐PTSD‐5) (Prins et al., 2016). Participants who reported the presence of a traumatic event in their lifetime responded yes/no to the five hallmark PTSD symptoms, such as trying to avoid memories of the trauma or situations associated with the event. Items are scored dichotomously (0 = *No*, 1 = *Yes*) and summed (range 0–5); scores ≥3 suggest probable PTSD (Prins et al., 2016). Traumatic events were also categorized according to trauma type based on a previously established typology of events (Kessler et al., 2017). The Cronbach's alpha of this measure in the current sample was .80.

#### Substance use

The 6‐item Alcohol, Smoking and Substance Involvement Screening Test for Youth (ASSIST‐Y) captured lifetime and past three month use of tobacco, alcohol, and cannabis (Humeniuk et al., 2016; WHO ASSIST Working Group, 2002). The ASSIST‐Y was adapted from the original version for adults and has been used to assess the substance use of South African adolescents (Sorsdahl et al., 2023). For each substance that participants reported lifetime use (yes/no), frequency of use in the past three months was rated from *Never* (0) to *Daily or almost daily* (6).

For each substance participants reported use of in the past three months, the level of risk was derived with the ASSIST‐Y as the sum of five scored items (questions 2‐6); for tobacco, question 5 is not scored (tobacco score consists of questions 2, 3, 4 and 6). ASSIST‐Y risk items use question‐specific weights rather than uniform anchors; item scores are summed for these items (possible range 0–30 for non‐tobacco substances and 0–22 for tobacco) to yield substance‐specific risk levels (low, moderate, or high). Moderate‐ and high‐risk categories indicate a level of use that negatively impacts functioning. Example questions to assess substance use risk include, “Has your use of (insert name of substance) led to problems with your health, relationships, finances, school, or with the police?” The standard guidelines for scoring and associated risk levels are substance and age dependent. These can be found in Table S2. In the present study, clinically relevant risk of substance use problems was defined as having a moderate or high risk of alcohol or cannabis use according to ASSIST‐Y scoring.

#### Treatment need awareness and access to care for mental health and substance use

Questions were adapted from the World Mental Health Surveys, which assess awareness of, and access to, mental health and substance use treatment in 21 countries, including five low‐and middle‐income countries (Alonso et al., 2018). Participants who reported at least one clinically relevant mental health or substance use problem (i.e., scored above the cut‐off on at least one measure), were asked whether, during the past 12 months, they felt that their condition required treatment, whether they had accessed care, and who provided the care. An example item from this questionnaire is, “In the past 12 months, have you felt that you needed professional treatment for the problems you shared with me about how you have been feeling?” Care provider options were as follows: specialized/general medical (e.g., psychiatric nurse, social worker, doctor, nurse), complementary alternative medical (e.g., traditional healer), non‐medical (e.g., religious or spiritual advisor), trained lay health worker (e.g., village health worker, lay counselor), and close family member, friend, or other community member without training.

#### Sweetened beverage consumption

Information on sweetened beverage consumption was assessed using two items from an 11‐item food frequency questionnaire, which was adapted from a South African obesity assessment tool to reflect local options in Lesotho (Okeyo et al., 2020). One item assessed the frequency of fruit beverage consumption and the other item assessed the frequency of soda consumption. Each item used a four‐point scale (1 = *None*, 2 = *Once or twice in the past week*, 3 = *Three to four times in the past week*, 4 = *Five or more times in the past week*). Responses were dichotomized across the two items to indicate any intake of either beverage (1 = *any intake of either beverage*) or none (0 = *no intake of either beverage*), consistent with evidence that fruit drinks and soda exert comparable mental health effects in adolescents (Pabayo et al., 2016).

#### Physical activity

Physical activity was assessed using the International Physical Activity Questionnaire Short Form (IPAQ‐SF; Craig et al., 2003). Participants were asked about the number of days (0–7) and minutes per day spent in vigorous activity (activities that require hard effort and make one breathe much harder), moderate activity (activities that require moderate effort and make it somewhat hard to breathe), and walking (including at work, at home, and traveling place to place) during the past 7 days. Responses were converted to Metabolic Equivalent of Tasks (METs) based on the frequency of each activity selected, following IPAQ scoring conventions. METs quantify the energy cost of physical activity, with 1 MET equal to the energy spent at rest. Participants were then classified as having a low, moderate, or high physical activity level.

#### HIV status

Due to the high prevalence of HIV in Lesotho, we asked participants to self‐report their HIV status. Options were HIV negative, HIV positive, or unknown/refused to answer.

### Data analysis plan

To describe the prevalence of mental health and substance use problems, frequencies and proportions of each problem were calculated according to standardized cut‐offs presented in the measures section. To examine developmental differences in mental health and substance use problem prevalence, descriptive results were disaggregated by age, representing early adolescence (age 10–14) and middle adolescence (age 15–17; WHO, 2006). Participant characteristics, mental health, and substance use were also disaggregated and descriptively compared by sex. Similarly, counts and proportions were reported to describe the awareness and treatment gaps.

To examine associations between modifiable risk factors (sugar‐sweetened beverage consumption and physical activity) and mental health and substance use outcomes, we constructed two multivariate logistic regression models to predict: (1) the presence of at least one symptom of a mental health problem or recent (past 3 months) use of a substance (alcohol or cannabis) and (2) the presence of any clinically relevant mental health (depression, suicidal ideation, anxiety, PTSD) or substance use (alcohol or cannabis) problem. We included symptoms of mental health conditions and recent substance use as one of our outcomes due to evidence that individual symptoms can significantly impair daily functioning (Karsten et al., 2010). Due to the low number of observed events in the sample (i.e., participants who had clinical levels of mental health or substance use problems), we combined levels of predictor variables when they were similar in strength and direction, such as moderate and high physical activity levels, to reduce the number of parameters and maintain an adequate events‐per‐variable ratio (roughly 8 per variable), thereby stabilizing estimates and preventing overfitting (Peduzzi et al., 1996). Multicollinearity between all model predictors (i.e., age, sex, sugar‐sweetened beverage consumption, and physical activity) was ruled out through the generalized variance inflation factor (Fox & Weisberg, 2019). All models were adjusted for sex and age group. Model parameters are presented as adjusted odds ratios (aOR), together with 95% confidence intervals (CI). Analyses were performed with R version 4.3.1 (R Core Team, 2021).

## RESULTS

### Prevalence of mental health and substance use problems

Overall, 3.0% of adolescents in the sample reported at least one clinically relevant mental health or substance use problem. Prevalence rates for anxiety symptoms, suicidal ideation, and use of all substances were higher among older adolescents aged 15–17 than younger adolescents aged 10–14. Prevalence rates for depressive and PTSD symptoms were higher among females, whereas prevalence rates for substance use were higher among males. Table 2 provides an overview of the prevalence rates for mental health and substance use problems for the overall sample and by age group. Sex‐specific prevalence rates for these conditions are provided in Table S3.

**TABLE 2:** Summary of clinically relevant depression, anxiety, post‐traumatic stress symptoms, and substance use problems overall and by age group.

Condition	Total	Age 10–14	Age 15–17
	*N* = 1351	*n* = 854	*n* = 497
	*n* (%)	*n* (%)	*n* (%)
Depressive symptoms			
Clinically relevant symptoms (≥10 score)	5 (0.3)	2 (0.2)	3 (0.6)
Mild symptoms (5–9 score)	28 (2.1)	8 (0.9)	20 (4.0)
Minimal symptoms (0–4 score)	1316 (97.6)	843 (98.7)	473 (95.2)
Missing	2 (0.1)	1 (0.1)	1 (0.2)
Suicidal thoughts	7 (0.5)	0 (0)	7 (1.4)
Missing	2 (0.1)	1 (0.1)	1 (0.2)
Anxiety symptoms			
Clinically relevant symptoms (≥6 score, South Africa cut‐off)	13 (1.0)	3 (0.4)	10 (2.0)
Clinically relevant symptoms (≥10 score, standard cut‐off)	3 (0.2)	0 (0)	3 (0.6)
Mild symptoms (5–9 score)	20 (1.5)	11 (1.3)	9 (1.8)
Minimal symptoms (0–4 score)	1326 (98.3)	842 (98.6)	484 (97.7)
Missing	2 (0.1)	1 (0.1)	1 (0.2)
Traumatic event, lifetime	62 (4.6)	27 (3.2)	35 (7.0)
Missing	3 (0.2)	2 (0.2)	1 (0.2)
Clinically relevant PTSD symptoms (≥3 score)^a^	12 (0.9)	8 (0.9)	4 (0.8)
Missing	3 (0.2)	2 (0.2)	1 (0.2)
Tobacco^a^, ^b^			
Lifetime use	24 (1.8)	5 (0.6)	19 (3.8)
Recent use (past 3 months)	20 (1.5)	3 (0.4)	17 (3.4)
Moderate or high risk	20 (1.5)	3 (0.4)	17 (3.4)
Missing	4 (0.3)	4 (0.5)	0 (0)
Alcohol^a^, ^b^			
Lifetime use	26 (1.9)	4 (0.5)	22 (4.4)
Recent use (past 3 months)	19 (1.4)	3 (0.4)	16 (3.2)
Low risk	6 (0.4)	N/A	6 (1.2)
Moderate or high risk	13 (1.0)	3 (0.4)	10 (2.0)
Missing	4 (0.3)	4 (0.5)	0 (0)
Cannabis^a^, ^b^			
Lifetime use	6 (0.4)	2 (0.2)	4 (0.8)
Recent use (past 3 months)	5 (0.4)	1 (0.1)	4 (0.8)
Moderate or high risk	5 (0.4)	1 (0.1)	4 (0.8)
Missing	4 (0.3)	4 (0.5)	0 (0)
Any clinically relevant mental health or substance use (alcohol or cannabis) problem	40 (3.0)	14 (1.6)	26 (5.2)
Any symptom of a mental health problem or recent use of alcohol or cannabis	426 (31.5)	226 (26.5)	200 (40.2)

Abbreviation: PTSD, post‐traumatic stress disorder.

^a^ Percentages represent the proportion of the overall sample.

^b^ There is no low‐risk use of tobacco or cannabis for ages 10–17 and no low‐risk use of alcohol among adolescents aged 10–14 based on the Alcohol, Smoking and Substance Involvement Screening Test for Youth (ASSIST‐Y) scoring.

### Treatment need and access to care

**FIGURE 1:**
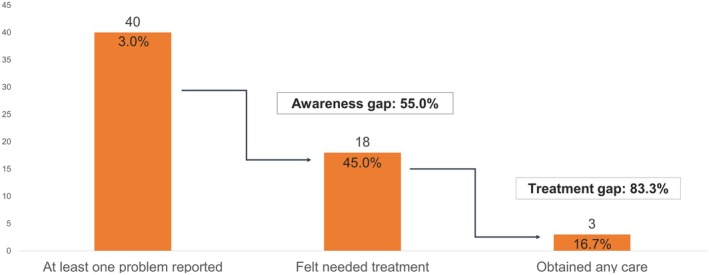
Cascade of care for participants with clinically relevant mental health or substance use problems.

Among those with clinically relevant mental health or substance use problems, 45% (18/40) were aware of the need for care; the vast majority of these participants (83% [15/18]) were female. Of those who were aware of the need for care, 56% (10/18) did not try to access care, while 17% (3/18) tried to access care but said support was unavailable, 11% (2/18) considered accessing support but did not follow through, 11% (2/18) accessed specialized or general medical support for their problem, and 6% (1/18) accessed care from a close friend or family member. Thus, out of 18 participants who were aware of a need for care, only three accessed treatment, resulting in a treatment gap of 83%. See Figure 1 for the treatment cascade among those who reported clinically relevant mental health or substance use problems.

### Association between lifestyle factors and mental health or substance use problems

When examining the relationship between lifestyle factors and symptoms of a mental health condition or recent substance use, consumption of sweetened beverages had a significant positive association with any symptom of a mental health condition or recent substance use (aOR: 2.29, 95% CI: 1.80–2.93), after adjusting for age, sex, and physical activity level. This indicates that the consumption of any sugar‐sweetened beverages was associated with an increased likelihood of having any symptom of a mental health condition or recent substance use. The consumption of sugar‐sweetened beverages remained significant in the model predicting clinically relevant mental health or substance use problems (aOR: 2.25, 95% CI: 1.13–4.48), after adjusting for age, sex, and physical activity level. Physical activity was not significantly associated with symptoms of a mental health condition/recent substance use or clinically relevant mental health or substance use problems. Table 3 provides full model results.

**TABLE 3:** Logistic regression models of the association between lifestyle factors and mental health or substance use problems.

Characteristic	Any mental health symptom or recent substance use aOR	95% CI	Clinically relevant mental health or substance use problems aOR	95% CI
Sex (male)	1.33	1.05, 1.70	1.38	0.72, 2.64
Age (15–17)	1.99	1.56, 2.56	3.39	1.72, 6.67
Sugar‐sweetened beverage consumption	2.29	1.80, 2.93	2.25	1.13, 4.48
Moderate or high physical activity	0.98	0.77, 1.25	0.92	0.48, 1.76

Abbreviations: aOR, adjusted odds ratio; CI, confidence interval.

## DISCUSSION

This study marks a first step in understanding mental health and substance use among adolescents in Lesotho using a population‐based survey based in two districts. Study findings show that 3% of the overall sample reported clinically relevant mental health or substance use problems. More female than male adolescents reported depressive and PTSD symptoms, while more older adolescents reported anxiety symptoms, as compared to younger adolescents. Moderate to high risk substance use was predominantly reported among male participants and older adolescents. The awareness and treatment gaps among adolescents with clinically relevant mental health or substance use problems were approximately 55% and 85%, respectively, suggesting that the majority of adolescents are unaware of the severity of their issues and do not access care.

Overall, the prevalence of clinically relevant mental health and substance use problems in this study was low, relative to most African estimates (Cortina et al., 2012; Jakobsson et al., 2024; Jörns‐Presentati et al., 2021; Ogundipe et al., 2018), but fell within the expected range based on global estimates (Bie et al., 2024; Institute of Health Metrics and Evaluation, 2021; Kieling et al., 2024; UNODC, 2024; WHO, 2018). A recent nationwide survey showed that adolescents aged 15–19 in Mokhotlong, the more remote of the two districts included in our study, reported the lowest prevalence of depression and anxiety symptoms in Lesotho (Ministry of Health Lesotho & USAID, 2024). Such findings, together with the similarly low prevalence we observed in neighboring Butha Buthe, may reflect communal resilience processes that remain strong in Lesotho's rural highlands. Research from similar settings across Africa shows that tight‐knit communal structures, extended family networks, and collective coping traditions buffer distress among adolescents (Rotzinger et al., 2025; Schwartz et al., 2017; Theron, 2023). While these mechanisms lay beyond the scope of the present survey, they point to the value of future research on indigenous protective systems, thereby advancing Marfo's (2016) call for worldview‐representativeness in adolescent developmental research.

Despite potential resilience in this population, service uptake among those with clinically relevant mental health or substance use problems was minimal. Of the 3% of youth who fell within this category , fewer than half recognized the need for treatment, and only 17% obtained any care. This gap parallels that among adults in Rwanda, where just 6% of those with mental health problems aware of services actually used them, and in rural Ethiopia, where only 13% of adults with alcohol use disorder sought help (Kayiteshonga et al., 2022). Additionally, a recent scoping review in six African countries estimated that only 10% of children and adolescents who need mental health care obtain it (Saade et al., 2023). Our cascade results align with the wider regional pattern of limited problem recognition and service use, outlining the need for combined demand‐and‐supply‐side strategies to narrow the treatment gap in Lesotho and other similar settings.

Developmental differences were observed in the study, with older adolescents aged 15–17 being three times more likely than their 10‐to‐14‐year‐old counterparts to meet clinical criteria for a mental health or substance use problem. This pattern is consistent with the Developmental Cascade Model of Adolescent Substance Use (Lynne‐Landsman et al., 2010), which predicts that early experimentation can snowball into heavier involvement as autonomy and peer influence grow (Mũrage et al., 2023; Profe & Wild, 2017). We also observed higher substance use risk among older male participants, which, taken together with evidence that one third of adult men in the same communities meet criteria for moderate‐to‐high risk alcohol use (Fernández, Yoon, et al., 2024), emphasizes the need for gender‐tailored prevention that begins in mid‐adolescence. Conversely, clinically relevant depressive and PTSD symptoms were more common among females, a finding that echoes broad literature showing steeper increases in internalizing problems for girls across diverse African and global contexts (Bie et al., 2024; Jakobsson et al., 2024; Osborn et al., 2020). At the same time, masculinity norms that discourage help‐seeking (Clark et al., 2020) may mask distress in males, suggesting that observed sex gaps could partly reflect under‐reporting. Together, these results point to distinct age‐ and gender‐specific leverage points for prevention and early intervention within Lesotho.

We found that sweetened beverage consumption—but not physical activity—was associated with the presence of any mental health symptoms/ recent substance use as well as clinically relevant problems. A likely reason for the non‐significant physical activity finding is the ceiling effect: nearly half of our adolescents fell in the IPAQ “moderate‐high” category, whereas studies of other African adolescents showed less physical activity (Asare & Danquah, 2015; Wachira et al., 2022). Rural youth in other African settings show high movement compared to urban youth. For instance, Kenyan adolescents in farming communities average 65 min of activity a day compared to 44 min of average daily movement for adolescents in Nairobi (Ojiambo et al., 2012), suggesting that routine chores, fieldwork, and long walking distances already embed substantial physical activity in daily life, leaving limited variability to detect mental health associations. Moreover, we only examined the first pillar of Ross' Framework of Adolescent Well‐Being, which specifically emphasizes good health and nutrition. Other pillars, such as safety, connectedness, and agency, may modulate the physical activity‐mental health connection in ways our study did not capture. Future studies in rural settings should pair physical activity measures with qualitative mapping of daily tasks to clarify intensity patterns and test whether physical activity benefits emerge once baseline movement and broader well‐being domains are considered.

## LIMITATIONS, FUTURE DIRECTIONS, AND IMPLICATIONS

This study's chief strengths are its large‐scale, population‐based design, which provides representative estimates of adolescent mental health and substance use problems in rural Lesotho, and its reliance on internationally recognized screening tools, which facilitate comparison with other settings. A further strength is the novel examination of modifiable lifestyle behaviors—rarely studied in low‐resource contexts—while the use of a predominantly rural sample is responsive to Marfo's (2016) call for the inclusion of diverse and more representative samples.

Study limitations include the use of assessment measures that have not been psychometrically tested with adolescents in Lesotho (although most were validated in neighboring South Africa), which may misclassify severity and underestimate prevalence. Because Western inventories may overlook culturally distinctive manifestations of distress (Haroz et al., 2017; Heim et al., 2022), future work should validate or indigenously develop measures that capture local phenomenology, aligned with decolonizing research principles (Marfo, 2016). Finally, because our data were drawn from only two predominantly rural districts, our findings are most generalizable to similar subsistence farming communities. Future research should replicate this study in multicountry rural cohorts to determine whether the same patterns hold across diverse agrarian contexts.

Overall, this study deepens our understanding of adolescent mental health and substance use in the predominantly rural setting of Lesotho. Even against a backdrop of relatively low prevalence, many adolescents who need help neither recognize that need nor obtain care. Targeted, adolescent‐responsive, and locally accessible strategies that simultaneously increase help‐seeking and expand the reach of services are therefore warranted. Future studies should continue to evaluate how modifiable lifestyle factors intersect with mental health and substance use problems, in addition to broader domains of well‐being across diverse rural contexts, so that evidence‐based policies can be tailored where healthcare infrastructure is weakest. Ultimately, expanding equitable access to care and identifying protective health behaviors are priorities to improve the well‐being of adolescents in rural communities so that they may enter adulthood with a stronger foundation for good mental health outcomes across the lifespan.

## AUTHOR CONTRIBUTIONS

Conceptualization—NEJ, RG, NDL, AA, JMB; data curation—NEJ, TTL; formal analysis—NEJ; funding acquisition—NDL, AA; investigation—NEJ, MPS; methodology—NEJ, JMB, FC, AA; project administration—MPS, RG; resources—RG, JMB, MPS; software—NEJ; supervision—JMB, AA, FC, NDL; validation—NEJ, FC; visualization—NEJ; writing—original draft—NEJ; writing—review and editing—all authors.

## FUNDING INFORMATION

The project is funded by the TRANSFORM grant of the Swiss Development Cooperation (project number 7F‐10345.01.01) and a grant by the World Diabetes Foundation (WDF‐1778). NDL’s salary was provided through the Swiss National Science Foundation Eccellenza grant (PCEFP3_181355). AA’s salary was provided through the Swiss National Science Foundation Postdoc mobility grant (P500PM_221961). JMB’s salary was provided through the Swiss National Science Foundation Ambizione grant (PZ00P1_201690). The funders had no role in the design of the study, the collection, analysis, and interpretation of data, or in the writing of the manuscript.

## CONFLICT OF INTEREST STATEMENT

The authors declare no competing interests.

## ETHICAL APPROVAL

This study was reviewed by the Ethics Committee of Northwest and Central Switzerland (ID AO_2021‐00056) and approved by the Lesotho Ministry of Health Research and Ethics Committee (ID 139‐2021) on October 8, 2021.

## PATIENT CONSENT

A caregiver provided written consent, and verbal assent was provided by the adolescent.

## PATIENT AND PUBLIC INVOLVEMENT

This survey is part of the Community‐based chronic care project Lesotho (ComBaCaL; www.combacal.org). It was designed together with the ComBaCaL steering committee that included a community representative, as well as representatives from the Ministry of Health of Lesotho. The survey was discussed with local authorities (village chiefs, local Ministry of Health), who were engaged throughout the survey.

## POSITIONALITY AND CULTURAL COMPETENCE

This study was conducted by an international, interdisciplinary team that brings together diverse social, cultural, and professional backgrounds. Several authors are affiliated with research institutions in Europe (NEJ, IFB, FC, FG, TTL, NDL, AA, and JMB) and have collaborated extensively in Lesotho on health research projects. Their familiarity with Lesotho stems from long‐term partnerships with local organizations over many years, with several authors (NDL, AA, FG and NEJ) also having lived in Lesotho. Four team members from Lesotho recruited and conducted the interviews with participants and were offered co‐authorship, with one agreeing to be a co‐author (MPS). Additional team members from Lesotho working on other health projects (RMj, RMk, JN, MM) and within the Ministry of Health’s Department of Mental Health (PL) ensured the cultural salience of the results. The analysis was completed by a PhD student (NEJ) originating from the United States with oversight from a statistician originating from Switzerland (FC).

## Supporting information


**Supplemental Table S1.** Sample characteristics overall and by sex.
**Supplemental Table S2.** Alcohol, smoking and substance involvement screening test for youth scoring.
**Supplemental Table S3.** Summary of clinically relevant depression, anxiety, post‐traumatic stress symptoms and substance use overall and by sex.

## Data Availability

The data that support the findings of this study are openly available in the Open Science Foundation at https://osf.io/j6avp/ reference number J6AVP.
