# Supplementary material for: Mental health and substance use problems among adolescents in Lesotho: Prevalence, access to care, and association with lifestyle factors
Source: J Res Adolesc. 2025 Aug 26;35(3):e70062. doi: 10.1111/jora.70062 (PMC12379077; doi:10.1111/jora.70062)
Supplement: Supplementary file 1 — Supplemental Table S1. Sample characteristics overall and by sex. Supplemental Table S2. Alcohol, smoking and substance involvement screening test for youth scoring. Supplemental Table S3. Summary of clinically relevant depression, anxiety, post‐traumatic stress symptoms and substance use overall and by sex. [file JORA-35-0-s001.docx]

**Supplemental Tables**

**Supplemental Table S1**

*Sample Characteristics Overall and by Sex*

|  | Total | Males | Females |
| --- | --- | --- | --- |
| Characteristic | N=1,351 (%) | n=665 (%) | n=686 (%) |
| Setting |  |  |  |
| Urban | 804 (59.5) | 382 (57.4) | 422 (61.5) |
| Rural | 547 (40.5) | 283 (42.6) | 264 (38.5) |
| Age (years) |  |  |  |
| 10-14 | 854 (63.2) | 423 (63.6) | 431 (62.8) |
| 15-17 | 497 (36.8) | 242 (36.4) | 255 (37.2) |
| Highest level of education completed |  |  |  |
| No schooling | 11 (0.8) | 8 (1.2) | 3 (0.4) |
| Primary school | 811 (60.0) | 430 (64.7) | 381 (55.6) |
| Secondary school | 525 (38.9) | 225 (33.8) | 300 (43.7) |
| Tertiary school^a^ | 4 (0.3) | 2 (0.3) | 2 (0.3) |
| Fruit beverage consumption in the past week |  |  |  |
| None | 781 (57.8) | 407 (61.2) | 374 (54.5) |
| Once or twice | 413 (30.6) | 187 (28.1) | 226 (32.9) |
| Three to four times | 93 (6.9) | 44 (6.6) | 49 (7.2) |
| Five or more times | 63 (4.6) | 27 (4.1) | 36 (5.3) |
| Missing | 1 (0.1) | 0 (0) | 1 (0.1) |
| Soda consumption in the past week |  |  |  |
| None | 1007 (74.5) | 513 (77.1) | 494 (72.0) |
| Once or twice | 297 (22.0) | 129 (19.4) | 168 (24.4) |
| Three to four times | 35 (2.6) | 17 (2.6) | 18 (2.6) |
| Five or more times | 11 (0.8) | 6 (0.9) | 5 (0.7) |
| Missing | 1 (0.1) | 0 (0) | 1 (0.1) |
| Physical activity level |  |  |  |
| Low | 681 (50.4) | 346 (52.0) | 335 (48.8) |
| Moderate | 497 (36.8) | 222 (33.4) | 275 (40.1) |
| High | 147 (10.9) | 84 (12.6) | 63 (9.2) |
| Missing | 26 (1.9) | 13 (2.0) | 13 (1.9) |
| Self-reported HIV status |  |  |  |
| Positive | 17 (1.3) | 10 (1.5) | 7 (1.0) |
| Negative | 543 (40.2) | 257 (38.6) | 286 (41.7) |
| Unknown | 789 (58.4) | 398 (59.9) | 392 (57.1) |
| Missing | 2 (0.1) | 0 | 2 (0.3) |
| Food shortage in the past month | 666 (49.3) | 329 (49.5) | 337 (49.1) |
| Missing | 3 (0.2) | 3 (0.4) | 0 |
| Number of people in the household, *Med (IQR)* | 3 (3-5) | 4 (3-5) | 3 (3-5) |
| Missing | 11 | 4 | 7 |

*Note.* Abbreviation: *Med*: median, *IQR*: interquartile range. *a* For adolescents aged 16–17 years, “tertiary school” reflects completion of a vocational training program. The survey question was phrased, “What is the highest level of education you have completed?” All respondents who selected this option were 16 or 17 years old.

**Supplemental Table S2**

*Alcohol, Smoking and Substance Involvement Screening Test for Youth Scoring*

| **Age group** | **Substance** | **Low-risk^a^** | **Moderate risk^a^** | **High risk** |
| --- | --- | --- | --- | --- |
| 10–14 y | Tobacco | N/A | 2–5 | ≥ 6 |
|  | Alcohol | N/A | 2–5 | ≥ 6 |
|  | Cannabis | N/A | N/A | ≥ 2 |
| 15–17 y | Tobacco | N/A | 2–11 | ≥ 12 |
|  | Alcohol | ≤ 4 | 5–17 | ≥ 18 |
|  | Cannabis | N/A | 2–11 | ≥ 12 |

*Note.* *A* There is no low-risk use of tobacco or cannabis for ages 10-17 and no low-risk use of alcohol among adolescents aged 10-14 based on the Alcohol, Smoking and Substance Involvement Screening Test for Youth (ASSIST-Y) scoring.

**Supplemental Table S3**

*Summary of Clinically Relevant Depression, Anxiety, Post-traumatic Stress Symptoms and Substance Use Overall and by Sex*

| **Condition** | **Total**  **N=1,351 (%)** | **Males**  **n=665 (%)** | **Females**  **n=685 (%)** |
| --- | --- | --- | --- |
| Depressive symptoms |  |  |  |
| Clinically relevant symptoms (≥ 10 score) | 5 (0.3) | 1 (0.1) | 4 (0.6) |
| Mild symptoms (5-9 score) | 28 (2.1) | 7 (1.1) | 21 (3.1) |
| Minimal symptoms (0-4 score) | 1316 (97.5) | 656 (98.7) | 660 (96.2) |
| Missing | 2 (0.1) | 1 (0.1) | 1 (0.1) |
| Suicidal thoughts | 7 (0.5) | 2 (0.3) | 5 (0.7) |
| Missing | 2 (0.1) | 1 (0.1) | 1 (0.1) |
| Anxiety symptoms |  |  |  |
| Clinically relevant symptoms (≥ 6 score, South Africa cut-off) | 13 (1.0) | 5 (0.8) | 8 (1.2) |
| Clinically relevant symptoms (≥ 10 score, standard cut-off) | 3 (0.2) | 2 (0.2) | 1 (0.1) |
| Mild, standard, cut-off (5-9 score) | 20 (1.5) | 5 (0.7) | 15 (2.2) |
| Minimal symptoms, cut-off (0-4 score) | 1326 (98.2) | 657 (99.0) | 669 (97.6) |
| Missing | 2 (0.1) | 1 (0.1) | 1 (0.1) |
| Traumatic event, lifetime | 62 (4.6) | 23 (3.5) | 39 (5.7) |
| Missing | 3 (0.2) | 1 (0.1) | 2 (0.3) |
| Clinically relevant PTSD symptoms (≥ 3 score)^a^ | 12 (0.9) | 1 (0.2) | 11 (1.6) |
| Missing | 3 (0.2) | 1 (0.1) | 2 (0.3) |
| Tobacco^a,b^ |  |  |  |
| Lifetime use | 24 (1.8) | 21 (3.2) | 3 (0.4) |
| Recent use (past 3 months) | 20 (1.5) | 18 (2.7) | 2 (0.3) |
| Moderate or high risk | 20 (1.5) | 18 (2.7) | 2 (0.3) |
| Missing | 4 (0.3) | 2 (0.3) | 2 (0.3) |
| Alcohol^a,b^ |  |  |  |
| Lifetime use | 19 (1.4) | 15 (2.3) | 11 (1.6) |
| Recent use (past 3 months) | 6 (0.4) | 13 (2.0) | 6 (0.9) |
| Low risk | 13 (1.0) | 4 (0.6) | 2 (0.3) |
| Moderate or high risk | 19 (1.4) | 9 (1.4) | 4 (0.6) |
| Missing | 4 (0.3) | 2 (0.3) | 2 (0.3) |
| Cannabis^a,b^ |  |  |  |
| Lifetime use | 6 (0.4) | 6 (0.9) | 0 |
| Recent use (past 3 months) | 5 (0.4) | 5 (0.8) | 0 |
| Moderate or high risk | 5 (0.4) | 5 (0.8) | 0 |
| Missing | 4 (0.3) | 2 (0.3) | 2 (0.3) |
| Any clinically relevant mental health or substance use (alcohol or cannabis) problem | 40 (3.0) | 16 (2.4) | 24 (3.5) |
| Any symptom of a mental health problem or recent use of alcohol or cannabis | 426 (31.5) | 185 (27.8) | 241 (35.2) |

*Note.* Abbreviation: PTSD=post-traumatic stress disorder. *a* Percentages represent the proportion of the overall sample. *b* There is no low-risk use of tobacco or cannabis for ages 10-17 and no low-risk use of alcohol among adolescents aged 10-14 based on the Alcohol, Smoking and Substance Involvement Screening Test for Youth (ASSIST-Y) scoring.
